# Targeting Src/FN1 adhesion circuitry to suppress circulating tumor cell clusters and lung cancer metastasis

**DOI:** 10.1016/j.gendis.2026.102154

**Published:** 2026-03-21

**Authors:** Ping Lin, Jianxin Jiang, Min Wu

**Affiliations:** Biological Science Research Center, Integrative Science Center of Germplasm Creation in Western China (Chongqing) Science City, Chongqing Technology Innovation Center of Breeding, Academy for Advanced Interdisciplinary Studies, Southwest University, Chongqing 400715, China; Wound Trauma Medical Center, State Key Laboratory of Trauma, Burns and Combined Injury, Daping Hospital, Army Medical University, Chongqing 400042, China; Wenzhou Institute, University of Chinese Academy of Sciences, Wenzhou 325001, China; Tianfu Jincheng Laboratory, Chengdu, Sichuan 610000, China; Department of Medicine, Harvard Medical School, Brigham and Women's Hospital, Boston, MA 02115, USA

## Background

**Figure 1:**
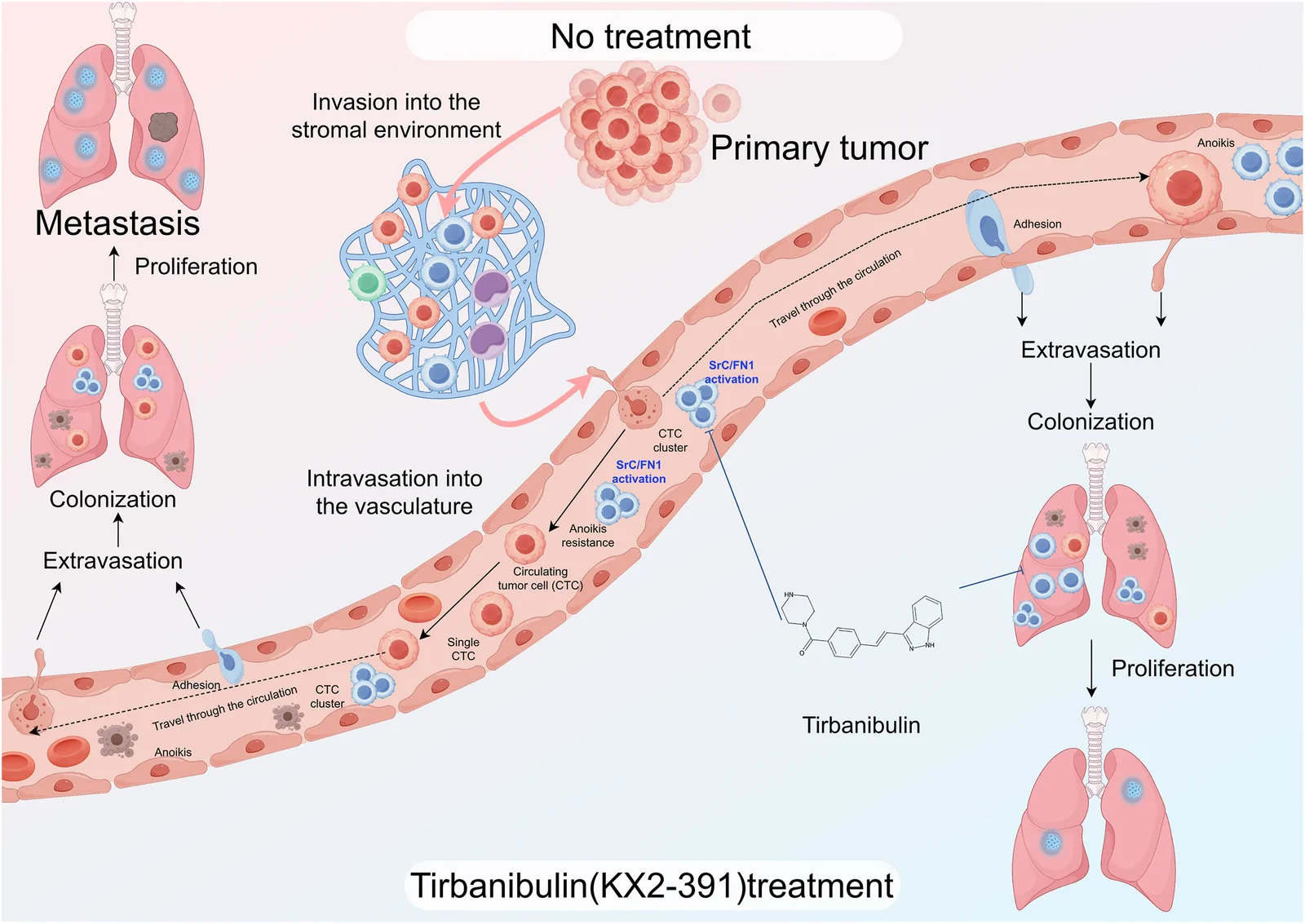
Conceptual schematic of adhesion-driven circulating tumor cells (CTCs) cluster metastasis and its disruption by Src inhibition. (Left Panel, No treatment): Single CTCs are sensitive to shear stress in the peripheral circulation and typically undergo anoikis. In contrast, CTC clusters activate the Src/FN1 pathway. This activation confers resistance to anoikis, enhancing their survival and increasing their ability to colonize the lungs and form metastases. (Right Panel, Tirbanibulin (KX2-391) treatment): Treatment with the Src inhibitor Tirbanibulin (KX2-391) blocks the Src/FN1 pathway. This inhibition prevents the formation of CTC clusters, restores anoikis sensitivity, and consequently impairs CTC survival, lung colonization, and ultimately the formation of metastatic lesions.

A recent paper published in *Science Advances* shows that circulating tumor cell (CTC) clusters—and not single CTCs—are the dominant drivers of recurrence and early mortality after surgery (Fig. 1). CTC clusters survive longer in circulation, lodge more tenaciously in lung microvasculature, and seed an eightfold excess of metastatic foci compared to single CTCs.^1^ These findings directly address the clinical challenge of postoperative recurrence, which affects half of resected non-small-cell lung cancer (NSCLC) patients within three years.

## Mechanistic insights

Clinically, lung cancer remains the leading cause of cancer-related death, with metastasis—not primary tumor burden—critically responsible for fatalities; however, the mechanisms underlying the metastasis development remain largely unknown.^2^ Through integrated transcriptomic and proteomic datasets, Que et al identify convergent upregulation of Src kinase activity and fibronectin-1 (FN1) expression within CTC clusters. The co-localization of phospho-Src and FN1 in cluster-forming cells underscores their functional interplay in cell–cell adhesion and mechanical protection. Genetic depletion of Src (via siRNA) or pharmacological inhibition with KX2-391 destabilizes cluster integrity without impairing cell viability, indicating that Src/FN1 drives cohesion rather than proliferation. This adhesive model echoes previous breast cancer CTC studies, in which adhesion molecules, such as CD44 and plakoglobin, promote collective dissemination,^3^^,^^4^ but uniquely define Src/FN1 as the “molecular Velcro” of lung cancer. Src/FN1 activation may endow CTC leaders with adhesive and mechanical advantage, enabling stable cohesion of clusters during vascular transit.

## From mechanistic insight to translational opportunity

KX2-391, a first-in-class oral substrate-site Src inhibitor with prior clinical testing, disrupts Src phosphorylation, diminishes FN1 levels, and physically dissociates clusters at sub-micromolar concentrations. In mouse models, administration of KX2-391 (4 mg/kg) reduces lung colonization by CTC clusters by 84% without systemic toxicity. Concordant decreases in Src/FN1 immunostaining in metastatic nodules validate target engagement. Importantly, Src/FN1 levels in primary tumors correlate inversely with overall survival in NSCLC cohorts, supporting their value as prognostic biomarkers and stratification tools for adjuvant therapy. Together, these results raise the prospect of a perioperative adjuvant strategy—short-term Src inhibition immediately after surgery to eradicate residual CTC clusters when the tumor burden is the lowest and immune surveillance remains intact. This approach could facilitate real-time monitoring of Src/FN1-positive clusters, guiding perioperative therapy with unprecedented precision.

## Conceptual and technical innovations

This work redefines metastasis as a collective, adhesion-driven phenomenon rather than a purely proliferation-driven process. The identification of Src/FN1 as “molecular Velcro” establishes a new therapeutic axis. Methodologically, Que et al’s suspension-based culture system to model the non-adherent state of CTCs in circulation, combined with orthogonal omics and *in vivo* validation, provides a versatile platform for interrogating rare circulating populations and screening anti-adhesion therapeutics. This framework has broad applicability across cancer types where collective dissemination is implicated. This aligns with the broader trend toward studying CTCs not just as biomarkers, but also as functional metastatic precursors amenable to *ex vivo* culture, CRISPR-based functional screens, and organoid modeling.

## Clinical and research implications

Clinical translation could follow several routes. A perioperative adjuvant strategy is particularly compelling: transient Src inhibition in the immediate postoperative window may neutralize residual CTC clusters when tumor burden is minimal and immune surveillance remains relatively intact. Biomarker-guided patient selection could be achieved by quantifying Src/FN1 in resected tumors—or via liquid biopsy—to enable risk-adapted therapy and predictive rather than empirical use of Src inhibitors. Finally, rational combinatorial therapy is warranted: although Src inhibitors exert limited impact on proliferation, they may synergize with cytotoxics or immune checkpoint inhibitors by simultaneously targeting disseminative adhesion circuits and proliferative tumor cells.^5^ Notably, PD-L1 expression has been detected on CTCs in NSCLC and other cancers, with variable predictive value for immunotherapy response. This raises the possibility of dual biomarker strategies—Src/FN1 for adhesion vulnerability and PD-L1 for immune modulation.

## Limitations and future directions

Despite a promising study, several questions remain unresolved. The authors may need to address certain limitations, such as the potential presence of drug-tolerant persister cells and the inherent constraints of small-animal models, which could influence the translational relevance of the findings. It would also be helpful for the authors to clarify which conclusions are directly supported by their data and which represent broader interpretation. What upstream triggers activate Src/FN1 specifically in cluster-forming CTCs? Does inhibition of Src/FN1 alter immune interactions during vascular transit or extravasation? Could intermittent perioperative dosing achieve durable suppression of early metastatic seeding while preserving wound healing? Finally, can detection of Src/FN1-positive clusters through liquid biopsy be developed into a clinically deployable companion diagnostic?

## Conclusion

Que et al provide compelling mechanistic and translational evidence that the Src/FN1 axis governs CTC cluster cohesion and metastatic spread in NSCLC. By positioning adhesion—not proliferation—as the key vulnerability, this study reframes metastasis as an adhesion-centric event and identifies transient Src inhibition as a credible therapeutic opportunity. It embodies a rare synergy between conceptual innovation and translational potential, highlighting a paradigm with applicability across the malignancies where collective tumor dissemination dictates clinical outcomes.

## CRediT authorship contribution statement

**Ping Lin:** Writing – review & editing, Writing – original draft, Funding acquisition, Conceptualization. **Jianxin Jiang:** Writing – review & editing, Writing – original draft, Funding acquisition, Conceptualization. **Min Wu:** Writing – review & editing, Writing – original draft, Funding acquisition, Conceptualization.

## Funding

This research was funded by the National Natural Science Foundation of China (No. 82470005, 82020108021) and the Key Research and Development Grant of MOST (China) (No. 2023YFA095000). This work was also supported by Chongqing Outstanding Youth Funds (China) (No. CSTB2025NSCQ-JQX0002) and the National Natural Science Foundation of Chongqing (China) (No. cstc2024ycjh-bgzxm0076), and a startup fund from Wenzhou Institute of University of Chinese Academy of Sciences (China) (No. WIUCASQD2022020).

## Conflict of interests

The authors declare that they have no conflict of interests.
